# Synthesis and Properties of Copolyphenylene Sulphones with Cardo Fragments

**DOI:** 10.3390/polym13213689

**Published:** 2021-10-26

**Authors:** Zhanna Kurdanova, Azamat Zhansitov, Kamila Shakhmurzova, Azamat Slonov, Artur Baykaziev, Svetlana Khashirova

**Affiliations:** Progressive Materials and Additive Technologies Center, Kabardino-Balkarian State University Named after H.M. Berbekov, St. Chernyshevsky, 173, 360004 Nalchik, Russia; kurdanova09@mail.ru (Z.K.); shahmurzova.kamila@yandex.ru (K.S.); azamatslonov@yandex.ru (A.S.); 2303tzoo@mail.ru (A.B.); new_kompozit@mail.ru (S.K.)

**Keywords:** polyphenylene sulfone, cardo fragments, IR spectroscopy, heat resistance, thermostability

## Abstract

Copolymers based on 4,4′-dihydroxydiphenyl, phenolphthalein and 4,4′-dichlorodiphenyl sulfone were synthesized by the method of high temperature polycondensation. The structure of the synthesized copolymers was confirmed by IR spectroscopy. Their physical, mechanical and thermal properties were investigated. It is shown that increases in the content of carded fragments lead to higher glass transition temperatures and heat resistance of the copolymers, as well as higher elastic and strength properties.

## 1. Introduction

Aromatic polysulfones are of great interest for various industries due to their particular set of valuable properties, such as heat resistance, fire resistance, high strength, etc. [1]. In order to expand the brand range of polysulfones, it is important to synthesize copolymers with both statistical and block structures [1].

Back in the thirties of the last century, the use of 4,4′-dioxyphthalophenone (phenolphthalein) as a bisphenol component in the synthesis of polyesters was proposed [2]. The first mention in the patent literature of the synthesis of polysulfones based on 4,4′-dioxyphthalophenone and 4,4′-dichlorodiphenylsulfone dates back to 1969 [3]; attention was mainly paid to the specific influence of cardo fragments on the properties of the polymer.

The introduction of cardo fragments into the structure of simple and complex aromatic polyesters gives them a number of specific properties. References [4,5]: an increase in the glass transition temperature; high values of heat resistance, which allow the copolymers to be used at higher temperatures without changing their mechanical properties; and the presence of bulky groups improves the solubility of polymers in strongly polar solvents, which makes it possible to process polymers from solutions. Thus, polymers with cardo groups, in addition to high thermal and physical-mechanical properties, are also characterized by a high transparency index and a high refractive index, along with low birefringence [6].

Studies have been carried out [7] on the effect of cardo groups on the dielectric properties of polyetherimides (PEI). These PEI-based films are characterized by good dielectric properties, with an ultra-low dielectric constant of 2.3 at 1 MHz, due to an increase in the free volume in the polymer chain. These materials are used as functional materials for the microelectronic, aerospace, electrical insulating and semiconductor industries. Another promising area developed over the last decade is the synthesis of polymer membranes used as gas separation systems and fuel cells [8,9]. The presence of cardo groups in the polymer membrane significantly affects their gas permeable characteristics due to the packing density and segmental mobility of the polymer chain. [10]. The authors of [11] cited studies on the gas barrier properties of membranes based on polyethersulfone (PES) and cardo polyethersulfone (PES-C)— there is an increase in selectivity with respect to N_2_, which increased from 2.95 to 4.24 barrers.; O_2_ permeability increased from 0.62 to 1.78 barrers and CO_2_ permeability increased from 6.87 to 13.80 barrers.

In subsequent years, research in the field of carded polyesters has been extensively developed [12,13,14,15,16]. Moreover, most of the available scientific publications are devoted to the study of the synthesis and properties of cardo polyethersulfones based on 4,4′-dihydroxydiphenylsulfone, 4,4′-dioxyphthalophenone and 4,4′-dichlorodiphenylsulfone [17,18,19,20,21,22]. Copolymers with cardo fragments based on 4,4′-dihydroxydiphenyl are mentioned in the patent technical literature without a detailed description of their physicochemical properties [23,24].

Earlier, in work [25], we reported on preliminary studies of the synthesis of cardo polyphenylene sulfones, with a content of 4,4-dihydroxyphthalophenone up to 50%. This article presents the results of a study of the whole series of copolymers of cardo polyphenylene sulfones synthesized by us in order to establish the dependence of the properties of the copolymer on the content of 4,4-dihydroxyphthalophenone.

## 2. Materials and Methods

### 2.1. Materials

4,4′-dihydroxy diphenyl and 4,4′-dichlorodiphenyl sulfone were kindly supplied by Alfa Aesar (Heysham, UK). Potassium carbonate and 4,4′-dihydroxy phthalophenone were purchased from Reachem (Moscow, Russia). N,N-dimethyl acetamide was purchased from Acros Organics (Geel, Belgium). N,N-dimethylacetamide (DMAc) was purified by distillation over calcium hydride and stored over 4-A° molecular sieves. All other reagents for the study were commercially obtained and used as received without further purification.

### 2.2. Characterization and Methods

The IR spectra of polymer samples in the form of a powder were recorded on a Fourier spectrometer Spectrum Two (PerkinElmer, Inc., Waltham, MA, USA), in the range of 4000–450 cm^−1^ with a spectral resolution of 0.4 cm^−1^.

The thermal properties of the polymers were investigated by differential scanning calorimetry using a DSC 4000 (PerkinElmer, Inc., Waltham, MA, USA), and by thermogravimetric analysis at a heating rate of 5 °C min^−1^ in an air atmosphere on a TGA 4000 instrument (PerkinElmer, Inc., Waltham, MA, USA).

The melt flow index (MFI) was determined on a capillary viscometer with IIRT-5 (Moscow, Russia) at a temperature of 350 °C and a load of 5 kg.

The reduced viscosity (η) of the synthesized polymers was determined in an Ubbelohde type capillary viscometer at 25 °C for 0.5 g of polymer in 10.0 mL of chloroform.

Mechanical tests were carried out on a universal testing machine, Gotech Testing Machine CT-TCS 2000 (Taichung, Taiwan) at 23 °C. Impact tests were performed with and without notch, by the Izod method on the instrument Gotech Testing Machine, Model GT-7045-MD (Taichung, Taiwan), with the energy of a pendulum, 11 J.

The Vicat softening temperature of the samples was determined on an HV-2000-M3 W Computer HDT/VICAT Tester Gotech Testing Machine (Taichung, Taiwan).

### 2.3. Synthesis of Polymers

#### 2.3.1. Synthesis of PPSU

Synthesis of polyphenylene sulfones (PPSU) was carried out in a 500 mL three-necked flask equipped with a nitrogen inlet, a mechanical stirrer, a Dean–Stark trap, and a reflux condenser; 4,4′-dihydroxy diphenyl (55.86 g, 0.3 mol), 4,4′-dichlorodiphenyl sulfone (89.16 g, 0.31 mol), and potassium carbonate (51.82 g, 0.375 mol) were charged in a flask. Then, N,N-dimethyl acetamide (470 mL) was added as reaction solvent. The reaction mixture was gradually heated to 165 °C for 4 h to distill the water. After the temperature reached 165 °C, the reaction mixture was allowed to proceed at this temperature for 6 h. After synthesis, the mixture was discharged and the formed salts were filtered. Then, the reaction solution was slowly poured into the water acidified by oxalic acid. The precipitated polymer was filtered and washed several times with water and dried in a vacuum oven at 160 °C for about 12 h.

#### 2.3.2. Synthesis of PPSU-C

As an example of the synthesis of copolyphenylene sulfone with cardo fragments, the synthesis with 30% phenolphthalein content is given. In the preparation of copolymers, PPSU with phenolphthalein as starting monomers in a flask were charged with 4,4′-dihydroxy diphenyl (39.1 g, 0.21 mol), 4,4′-dihydroxy phthalophenone (phenolphthalein) (28.65 g, 0.09 mol), 4,4′-dichloro diphenyl sulfone (89.16 g, 0.31 mol), and potassium carbonate (51.82 g, 0.375 mol). Further synthesis was carried out as described above.

## 3. Results and Discussion

**Scheme 1:**
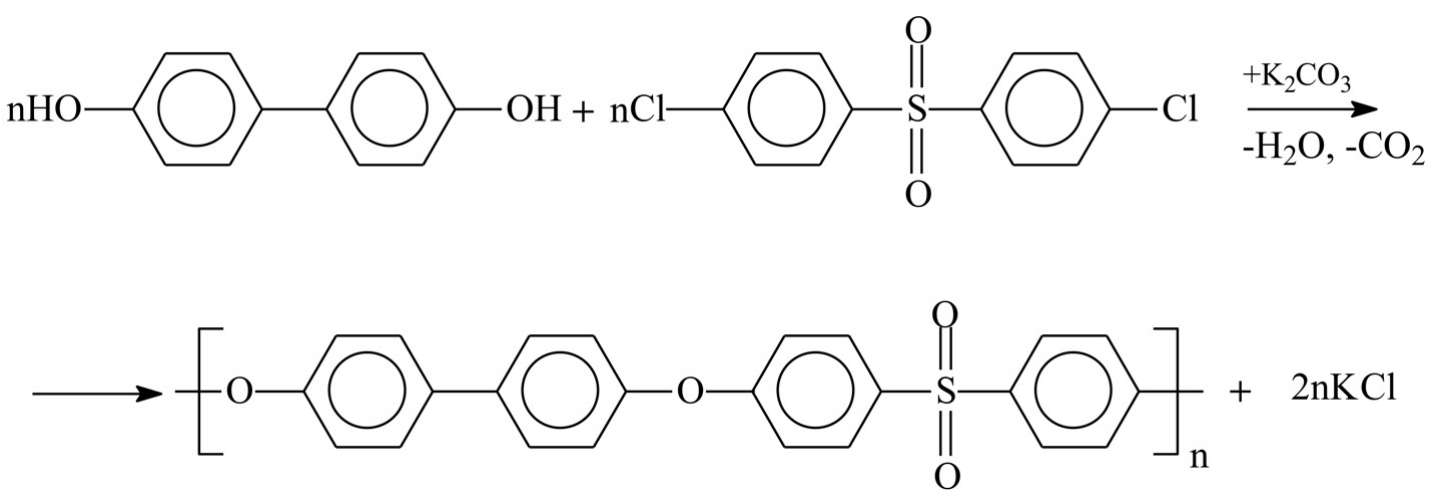
Synthesis of PPSU.

**Scheme 2:**
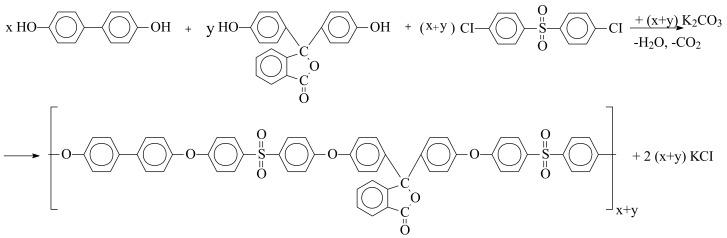
Synthesis of copolymer PPSU with phenolphthalein.

The synthesis of copolyphenylene sulfones was carried out by high-temperature polycondensation by the mechanism of a nucleophilic substitution reaction. PPSU was synthesized according to Scheme 1; a copolymer of polyphenylene sulfone with 4,4′-dioxyphthalophenone was synthesized according to Scheme 2.

**Figure 1:**
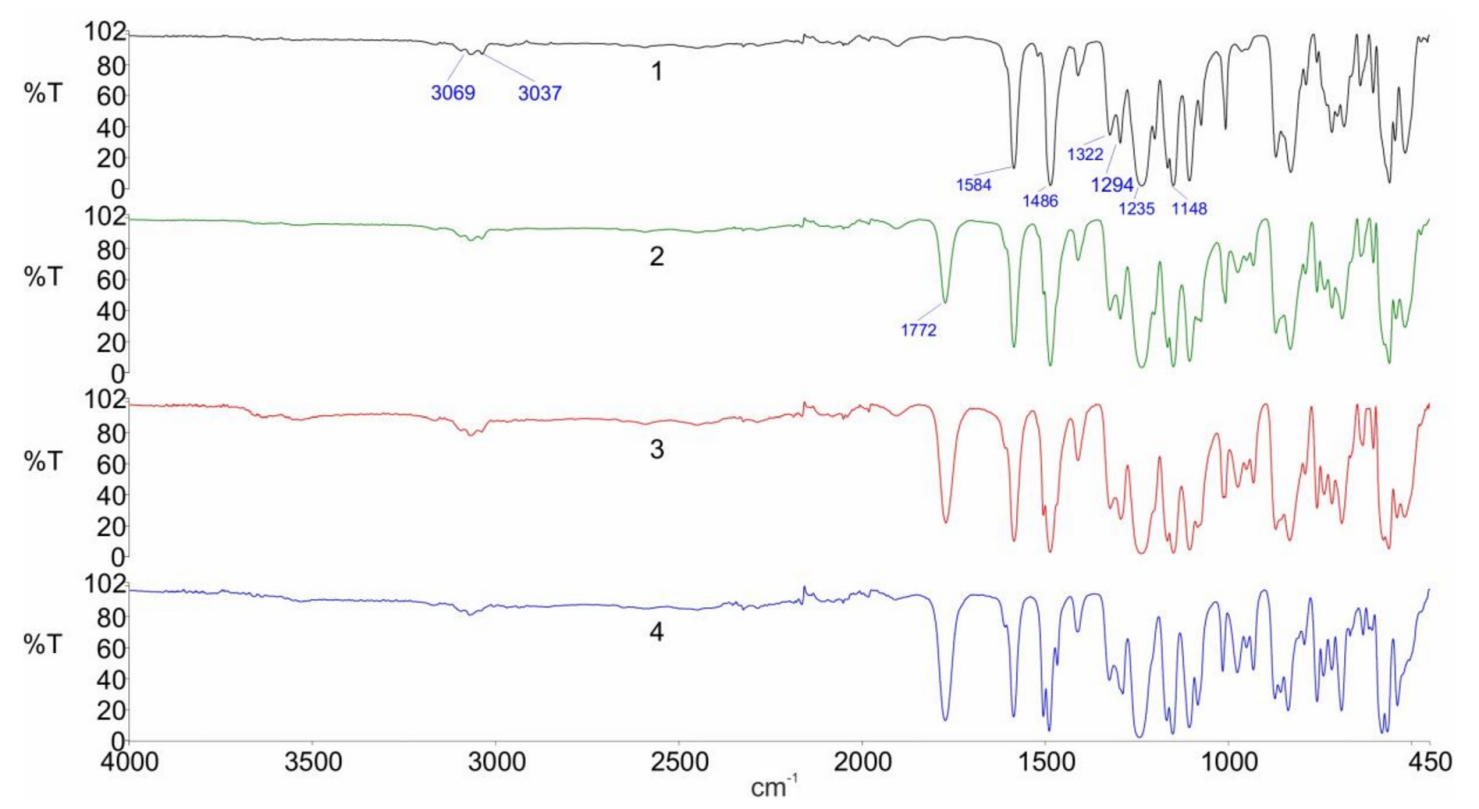
IR spectra: 1—PPSU; 2—PPSU-C-30; 3—PPSU-C-50; 4—PPSU-C-100.

The structure of the synthesized copolymers was studied by IR spectroscopy. In the spectra of synthesized PPSU, absorption bands due to stretching vibrations of C–H bonds were characterized by a weak intensity, and appeared as two peaks with maxima at 3037 and 3066 cm^−1^ (Figure 1).

Skeletal vibrations of aromatic C–C bonds are manifested in bands with maxima at 1584 and 1486 cm^−1^. Polyphenylene sulfones also have a very characteristic absorption in the range of 1350–1300 cm^−1^ and 1170–1120 cm^−1^, caused by symmetric and antisymmetric vibrations of the SO_2_ group, respectively. In the spectra of the copolymers synthesized in this work, similar symmetric and antisymmetric vibrations of the SO_2_ group appeared as a split band with peaks at 1322 and 1294 cm^−1^, as well as a second split band with peaks at 1165 and 1148 cm^−1^, respectively. An intense band at 1235 cm^−1^ is associated with asymmetric stretching vibrations of the C–O group [1,26].

The main distinguishing feature confirming the presence of the phenolphthalein component in the copolymers is the presence of a carbonyl group, which appears as a band with a maximum at 1772 cm^−1^. In this case, the intensity of this band could also be judged on the percentage of 4,4′-dioxyphthalophenone in the copolymers.

**Scheme 3:**
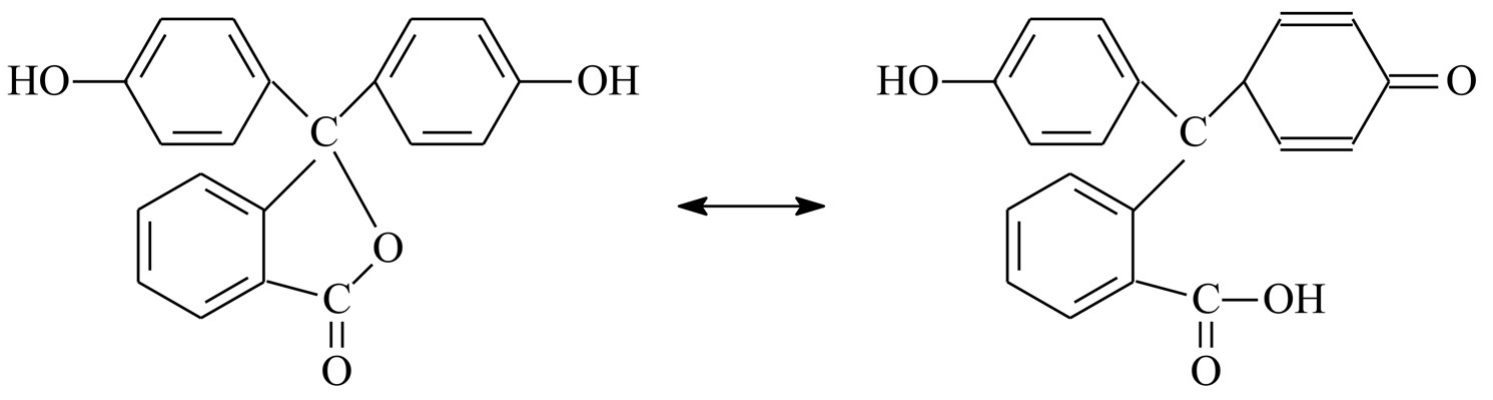
Tautomeric rearrangement in the 4,4′-dihydroxyphthalophenone molecule.

As can be seen from the data shown in Table 1, with an increase in the content of 4,4′-dihydroxyphthalophenone at the same temperature–time modes of synthesis, the reduced viscosity of the samples decreased, which is associated with the lower reactivity of 4,4′-dihydroxyphthalophenone compared to 4,4′-dihydroxydiphenol. With a strictly equivalent ratio of monomers, the final molecular mass of the polymer was determined by the acid–base properties of the monomers, the ratio of the rates of the main chain growth reaction and the side reactions limiting the chain growth. Due to the higher acidity of the cardo monomer, a side reaction occurs during polycondensation—the formation of a quinoid structure as a result of a tautomeric rearrangement in the 4,4′-dihydroxyphthalophenone molecule [27], according to the following Scheme 3:

In this case, the value of the MFI was also decreased by an increase in the fraction of cardo fragments of 4,4′-dihydroxyphthalophenone. This is presumably caused by the hindrance of bulky side substituents on the movement of macro molecules relative to one another in the melt.

One of the most important indicators in the use of high performance polymers is a glass transition temperature, which is directly related to the maximum possible temperature of their long-term operation.

It is known [1,28] that the introduction of large side substituents into the polymeric chain increases the heat resistance of the material. As expected, the introduction of phenolphthalein significantly increased the glass transition temperature and heat resistance (Table 2).

**Figure 2:**
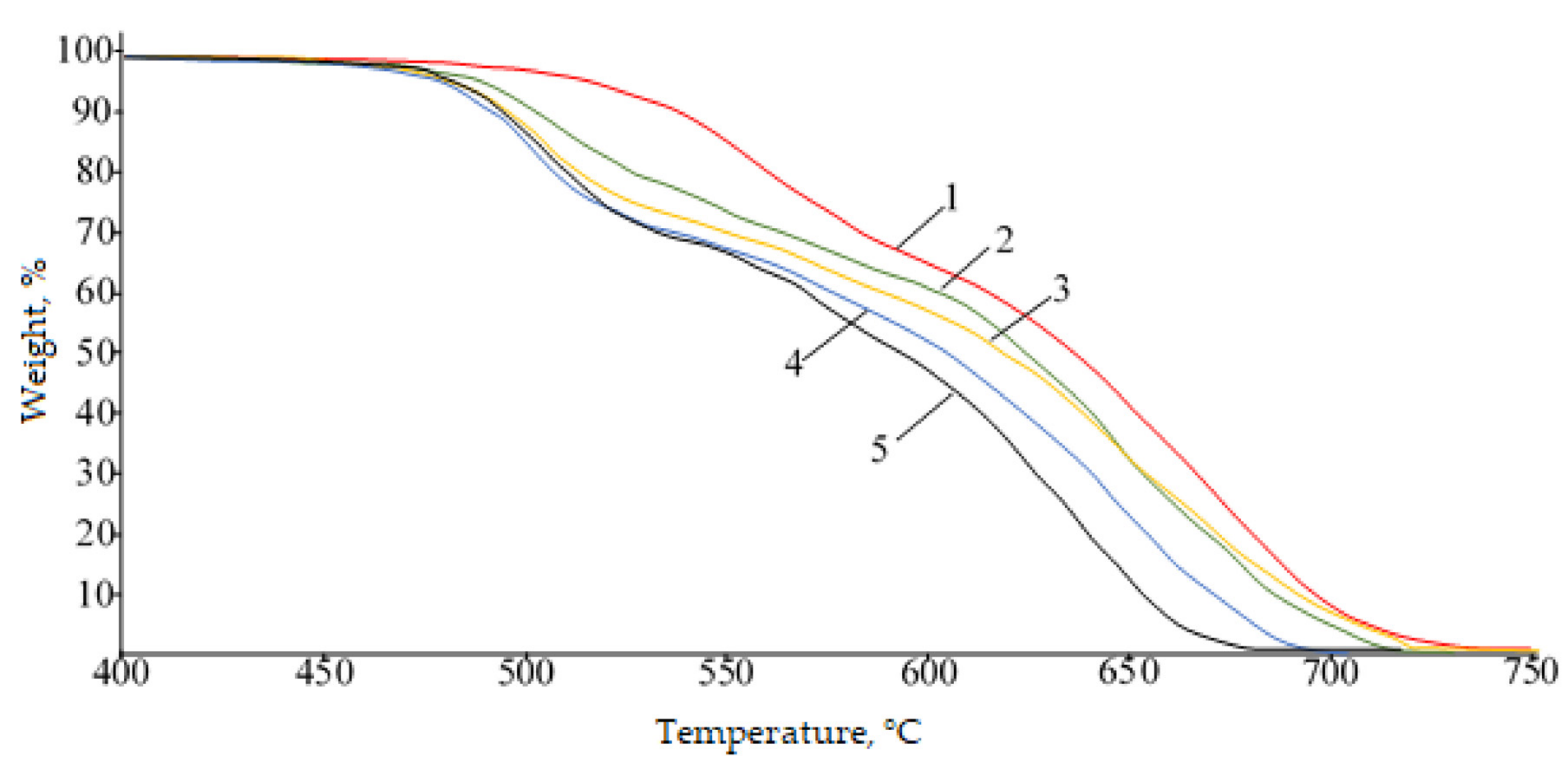
TGA curves: 1—PPSU; 2—PPSU-C-30; 3—PPSU-C-50; 4—PPSU-C-70; 5—PPSU-C-100.

The temperatures at the beginning of the mass loss of the synthesized copolymers in air, as can be seen from the results presented in Table 2 and Figure 2, insignificantly decreased with an increase in the concentration of cardo fragments. Thus, the addition of 10% 4,4′-dihydroxyphthalophenone reduced the temperature of the onset of thermal degradation, corresponding to a loss of 2% of the mass, by only 13 °C, and the addition of 50% 4,4′-dihydroxyphthalophenone reduced the heat resistance by 26 °C. Further, with an increase in the content of the proportion of cardo fragments, the temperature of the onset of thermal degradation practically did not change.

The main physical and mechanical properties of copolyphenylene sulfones with cardo fragments of 4,4′-dihydroxyphthalophenone are shown in Table 3.

As can be seen from Table 3, there is a direct dependence of the elastic-strength properties on the 4,4′-dihydroxyphthalophenone content: with an increase in its concentration, the elastic modulus, both in bending and in tension, gradually increased (by about 14% at 50% comonomer content). These results are consistent with the nature of the change in Tg, which characterizes the rigidity of the polymer chain. Strength characteristics also tended to increase. When the comonomer content was above 50%, the fracture became brittle, without plastic flow—which explains the increased strength at a break at 70% content of 4,4′-dihydroxyphthalophenone; however, further, due to the decrease in reduced viscosity, the strength decreased. Clearly, the observed changes in properties are the result of an increase in the kinetic rigidity of the chain, as well as the creation of conditions for the “linkages” of structural elements due to the presence of a bulky substituent.

Additionally, copolymers have lower values of impact strength than homopolymers, which appears to be the result of two factors. First, toughness is highly dependent on molecular weight. As shown above (Table 1), the values of the average molecular weight of the copolymers (Mw) monotonically decreased with an increase in the concentration of 4,4′-dihydroxyphthalophenone, which, in turn, can lead to a loss of impact resistance. Secondly, it can be seen from Table 3 that even at a content of 10% comonomer, the impact strength and elongation in tension were significantly lower than the corresponding properties of the homopolymer, despite the close values of the rheological and molecular weight characteristics. This suggests that the presence of 4,4′-dihydroxyphthalophenone cardo fragments in itself already leads to a decrease in plastic properties.

Shore hardness studies of the synthesized polymers showed (Table 3) that an increase in the content of the proportion of 4,4′-dihydroxyphthalophenone cardo fragments above 70 mol.% did not lead to an increase in the hardness values of the samples

## 4. Conclusions

As a result of the studies carried out, the influence of the concentration of 4,4′-dioxyphthalophenone on the molecular weight characteristics of the obtained copolymers was revealed. Their structural features were confirmed by IR spectroscopy. It is shown that an increase in the content of cardo fragments led to an increase in the glass transition temperature and heat resistance of copolymers, as well as elastic-strength properties, due to an increase in chain rigidity. In this case, a decrease in heat resistance, as well as the plastic properties of the copolymers, was observed.

## Figures and Tables

**Table 1:** Rheological properties.

Sample	η (dL g^−1^)	MFI (g min^−1^)
PPSU	0.50	14.0
PPSU-C-10	0.47	13.0
PPSU-C-30	0.40	12.0
PPSU-C-50	0.31	9.0
PPSU-C-70	0.31	10.0
PPSU-C-90	0.30	4.3
PPSU-C-100	0.28	5.4

**Table 2:** Thermal properties of PPSU and PPSU-C.

Sample	T_2%_, °C	T_5%_, °C	T_10%_, °C	Tg, °C	Vicat Softening Temperature, °C
PPSU	490	517	538	219	221
PPSU-C-10	477	500	518	221	222
PPSU-C-30	470	489	504	229	229
PPSU-C-50	464	483	497	235	233
PPSU-C-70	462	480	493	241	239
PPSU-C-90	462	481	494	245	244
PPSU-C-100	465	482	494	248	248

T_2%,_ T_5%,_ T_10%:_ temperature of 2, 5 and 10% weight loss, respectively.

**Table 3:** Mechanical properties of PPSU and PPSU-C.

Sample	Impact Strength, kJ/m^2^		E_fl_,	σ_yield_,	E_ten_,	σ_ten_,	ε,	Shore Hardness (D Scale)
	Unnotched	Notched	MPa	MPa	MPa	MPa	%	
PPSU	n/b	24.3	2390	87.5	2150	73	38.5	75
PPSU-C-10	150	14.8	2500	83.0	2230	68	12.6	76
PPSU-C-30	168	11.0	2650	87.0	2300	70	11.7	78
PPSU-C-50	88	6.2	2780	90.5	2430	73	9.8	79
PPSU-C-70	27	6.5	2970	-	2613	94	8.0	80
PPSU-C-90	15	4.5	3110	-	2330	83	4.5	80
PPSU-C-100	26	9.8	3270	-	2490	85	4.2	80

E_fl_: flexural modulus, σ_yield_: Yield strength, E_ten_: tensile modulus, σ_ten_: tensile strength, ε: elongation at break. n/b: not break.
